# Mechanistic evidence and assumptions in mycotoxin-gut interaction

**DOI:** 10.3389/ffunb.2026.1855881

**Published:** 2026-08-17

**Authors:** Esther Garcia-Cela, Alessandra Marcon Gasperini

**Affiliations:** 1 MycoLab, School of Health, Medicine and Life Sciences, University of Hertfordshire, Hatfield, United Kingdom; 2 UCD School of Agriculture and Food Science, University College Dublin, Belfield, Dublin, Ireland

**Keywords:** antimicrobial resistance, dysbiosis, gut microbiota, host–microbiota interactions, intestinal barrier, microbial detoxification, mycotoxins, xenobiotics

## Abstract

Mycotoxins are increasingly recognised as ecological mediators that act through a bidirectional gut–microbiota axis. Microbial metabolism governs toxin fate by driving detoxification, bioactivation, sequestration and deconjugation, whereas mycotoxin exposure reshapes microbial communities, disrupts epithelial barrier function and amplifies immune signalling. Across aflatoxin B1, ochratoxin A, fumonisin B1, deoxynivalenol and T-2 toxin, the microbiome emerges as both a determinant and a target of toxicity, with strain-level functional diversity influencing susceptibility, detoxification capacity and host outcome. A further consequence of chronic exposure may be selection for pathobionts and antibiotic-resistant populations, raising the possibility that mycotoxins contribute to microbiome instability beyond direct toxic effects. Despite these advances, most evidence remains correlative or model-specific, and causal validation *in vivo* is still limited. Integrating microbiology, metabolomics, epithelial biology and ecological theory is essential to understand these interactions and to better guide microbiome-informed strategies for mycotoxin exposure mitigation.

## 1 Introduction

Fungi produce diverse secondary metabolites that enhance survival by mediating competition, environmental adaptation and inter-organismal interactions. These compounds appear from specialised biosynthetic systems, including polyketide synthases, non-ribosomal peptide synthetases and terpenoid pathways, and extend far beyond primary metabolism (Keller, 2019). Recent studies have reported more than 900 new fungal metabolites, predominantly polyketides, terpenoids and peptides (Shi et al., 2024; Bao et al., 2025). Among these, mycotoxins form a chemically diverse group that frequently contaminates food and feed and causes hepatotoxic, nephrotoxic, immunotoxic and carcinogenic effects in humans and animals (Milićević et al., 2010; García-Cela et al., 2012; Awuchi et al., 2021).

Although mycotoxins are conventionally defined by their toxicological effects, their production is increasingly understood as part of specific fungal ecological strategies, including competition with microorganisms, defence against herbivores and adaptation to environmental stress, rather than as adaptive toxicity toward higher organisms in general (Frisvad et al., 2018). For example, aflatoxin production increases the fitness of *Aspergillus flavus* by up to 26-fold against insect herbivores while suppressing competing soil microorganisms (Drott et al., 2017), and this trade-off persists in natural populations (Sweany et al., 2022). This ecological framing aligns with the broader therapeutic utility of fungal secondary metabolites, including antibiotics, antifungals and immunosuppressants, derived from the same biosynthetic machinery, suggesting that the distinction between mycotoxins and pharmaceuticals reflects producer organism and exposure context rather than chemical mechanism (Boruta, 2018).

The ecological and metabolic continuum of mycotoxins extends beyond ingestion, as dietary mycotoxins encounter the gut microbiota, a highly dynamic metabolic interface that shapes xenobiotic transformation and consequently modulates mycotoxin bioavailability, toxicity and host exposure (Gasperini et al., 2025; Li et al., 2025). As with pharmaceuticals, the gut microbiota modifies mycotoxin bioavailability and toxicity through enzymatic biotransformation and binding (Guerre, 2020), while mycotoxins reciprocally reshape microbial communities through direct antimicrobial effects, environmental modulation and host-mediated responses (Liew and Mohd-Redzwan, 2018; Xia et al., 2022a; Gasperini et al., 2025).

Over the past decade, bidirectional mycotoxin–microbiota interactions have been increasingly documented, yet mechanistic resolution remains limited. Microbial modification of dietary mycotoxins through conserved enzymatic and binding mechanisms has been reported across vertebrate and invertebrate taxa (Hou et al., 2025). Detoxification is exemplified by de-epoxidation of deoxynivalenol (DON) to the less toxic de-epoxy-deoxynivalenol (DOM-1), whereas bioactivation occurs when zearalenone (ZEN) is converted to the more estrogenic α-zearalenol (Guerre, 2020). In addition, microbial cell walls sequester mycotoxins, microbial deconjugation during enterohepatic cycling prolongs systemic exposure, and masked plant-derived mycotoxins can be hydrolysed back to active forms in species- and site-dependent ways (Daud et al., 2020; Guerre, 2020; Hou et al., 2025). However, many reports of mycotoxin-induced microbiota perturbation remain largely descriptive, with limited causal or functional resolution (Xia et al., 2022b). Collectively, these findings indicate that mycotoxin fate is shaped by microbiota-specific metabolic capacity rather than being intrinsically fixed (Guerre, 2020; Hou et al., 2025). In this perspective, we review evidence on mycotoxin–gut microbiota interactions, and we propose an ecological paradigm in which the microbiome functions as both a determinant and a target of mycotoxin toxicity. This framework is based on five interconnected principles: microbial metabolism governs mycotoxin detoxification, bioactivation and systemic exposure; mycotoxins act as ecological stressors that restructure microbial communities; microbiota-mediated barrier disruption and immune activation amplify host toxicity; chronic low-dose exposure may contribute to antimicrobial resistance evolution; and strain-level functional diversity determines detoxification capacity, resistance and pathogenic potential. This paradigm provides a mechanistic foundation for understanding mycotoxin biology and for developing microbiome-informed strategies to mitigate mycotoxin-associated risks.

## 2 Microbial processes that directly modify mycotoxin fate and toxicity in the gut

The gut microbiome modulates mycotoxin fate through conserved mechanisms that influence bioavailability, persistence, and biological activity (**Table 1**). These mechanisms include enzymatic biotransformation, which can result in detoxification or bioactivation; physical binding and sequestration by microbial cell structures; and microbial deconjugation reactions that can regenerate masked or host-conjugated toxins during enterohepatic cycling. The relative contribution of each pathway depends on the chemical properties of the toxin and microbiome composition and functionality.

**Table 1:** Bidirectional mycotoxin–microbiota–host interactions and mechanistic evidence across AFB1, OTA, FB1, DON, and T-2 toxin.

Mycotoxin	Effect on gut microbiota (dysbiosis & metabolism)	Microbial modification of mycotoxin	Host–microbiota interactions & causal validation	Ref.
AFB1	**Dysbiosis pattern:** ↑ *Bacteroides/Parabacteroides* ↓ barrier integrity LPS translocation **Metabolite shifts:** ↓ SCFAs, especially butyrate and propionate; ↑ pipecolic acid and norepinephrine. **ROS amplification:** dysbiotic microbiota enhance ROS via TLR4 priming, worsening epithelial damage.	**Probiotic binding:** *Lactobacillus*/*Bifidobacterium*cell-wall peptidoglycan adsorption with limited enzymatic degradation. **Bioavailability**: dysbiosis reduces microbial binding capacity, increasing free AFB1 absorption in the small intestine.	**Pathway**: Pipecolic acid and norepinephrine activate TLR4/NLRP3, causing splenic pyroptosis. ↑ IL-6, TNF-α, IL-1β; hepatic inflammation secondary to LPS. **Causal validation**: ABX eliminates splenic pyroptosis; FMT or sterile filtrate reproduces it. TLR4^−^/^−^ or NLRP3^−^/^−^ mice block pyroptosis despite persistent dysbiosis.	Sobral et al. (2022); Ye et al. (2023, Ye et al., 2024a); Chen et al. (2024a)
OTA	**Dysbiosis pattern:** ↓ beneficial taxa (*Lactobacillus* and *Faecalibacterium)* ↑ opportunistic pathogens and LPS producers (*B. plebeius* and *Prevotella)* **Metabolic disruption**: ↓ secondary bile acids via 7α-dehydroxylase reduction;n ↓ butyrate, acetate, and propionate, impairing FXR/TGR5 signalling. **Tryptophan depletion:** ↓ nicotinuric acid, affecting NAD^+^ and AMPK homeostasis.	**Hydrolysis and binding:** carboxypeptidase/peptidase cleavage to less toxic OTα. **Fermented food consortia, such as Tibetan kefir, show multimodal action**: luminal binding via β-glucans/peptidoglycans, enzymatic hydrolysis, and ↑ *Akkermansia* and *Lachnospiraceae*.	**Pathway**: Barrier disruption with ↓ ZO-1, ↓ claudins, leading to LPS translocation and hepatic TLR4 activation. ↓ NAD^+^ triggers maladaptive AMPK and liver injury. **Causal validation**: kefir intervention lowers OTA burden, restores tryptophan/NAD^+^, upregulates CLDN1/OCLN/TJP1, reduces ALT/AST, and metabolomics confirms glycerophospholipid rebalancing.	Taheur et al. (2017); Du et al. (2021, 2022); Ma et al. (2023); Więckowska et al. (2023)
FB1	**Dysbiosis signature:** Firmicutes/Bacteroidetes imbalance. ↓ *Faecalibacterium* ↑ Proteobacteria. **Regional injury:** caecal/colonic epithelial damage exceeds small intestine damage; ↓ mucus thickness. **SCFA depletion**: acetate, butyrate, and propionate reduced by 40–60% ↓ phenolic acids and indoles, compromising AhR signalling.	**Enzymatic hydrolysis:** fumonisin esterases/amidases, including from *Lactobacillus* spp., remove tricarballylic acid to form HFB1. **Regional metabolism:** cecum shows robust HFB1 accumulation, with higher hydrolysis; small intestine shows minimal hydrolysis.	**Immune/pathology:** - villus atrophy; MAPK/NF-κB activation drives ↑ IL-6, TNF-α, IL-1β, independent of ceramide synthase inhibition; hepatic steatosis and inflammatory infiltrates follow. **Causal validation:** caecal α-diversity loss correlates with liver pathology, FMT reproduces hepatic injury, and ABX eliminates 75–80% of hepatotoxicity.	Dantzer et al. (1999); Masching et al. (2016); Gu et al. (2019); Yu et al. (2022a); Dang et al. (2025); Ye et al. (2025)
DON and T-2	**DON/T-2 effects**: ↓ SCFAs; ↑ ROS and lipid peroxidation; ↓ barrier integrity with ↓ ZO-1 and claudins. **Anorexia pathway:** enteroendocrine modulation via GLP-1/PYY suppression, amplified by dysbiosis. **T-2 in poultry:** ↓ *Lachnospiraceae* IL-6/JAK/STAT1 activation in spleen.	**DON degradation**: *Bacillus velezensis* actively degrades DON in simulated GI fluid and maintains colonization in the cecum. *Lactobacillus rhamnosus* GG binds DON via S-layer protein; lipoteichoic acids enhance barrier signalling. **Deconjugation:** colonic microbiota releases toxic aglycones from masked DON-glucosides via β-glucosidases.	Ribotoxic stress activates MAPK/NF-κB, raising pro-inflammatory cytokines. DON/T-2 drive microbiota-dependent anorexia and splenic injury. **Causal validation:** ABX/FMT models show anorexia is microbiota-dependent; LGG rescues food intake, increases butyrate, and lowers TNF-α/IL-6; *B. velezensis* restores fermentation genes and reduces MDR efflux pump effects. For T-2, ABX abolishes splenic injury and FMT recapitulates it; nano-selenium suppresses IL-6/JAK/STAT1 via ROS reduction.	Wang et al. (2012, Wang et al., 2024); Lucke et al. (2018); Zhai et al. (2022); Bai et al. (2023); Liang et al. (2024); Liu et al. (2025); Huang et al. (2026); Jin et al. (2026)

Symbols ↑ and ↓ indicate an increase or decrease in abundance, expression, or concentration, respectively. AFB1, aflatoxin B1; OTA, ochratoxin A; OTα, ochratoxin alpha; FB1, fumonisin B1; HFB1, hydrolysed fumonisin B1; DON, deoxynivalenol; ABX, antibiotic treatment; FMT, fecal microbiota transplantation; SCFAs, short-chain fatty acids; LPS, lipopolysaccharide; ROS, reactive oxygen species; TLR4, Toll-like receptor 4; NLRP3, NOD-like receptor family pyrin domain-containing 3; NF-κB, nuclear factor kappa B; MAPK, mitogen-activated protein kinase; IL, interleukin; TNF-α, tumour necrosis factor alpha; FXR, farnesoid X receptor; TGR5, Takeda G protein-coupled receptor 5; AhR, aryl hydrocarbon receptor; NAD^+^, nicotinamide adenine dinucleotide; AMPK, AMP-activated protein kinase; GLP-1, glucagon-like peptide-1; PYY, peptide YY; ZO-1, zonula occludens-1; CLDN1, claudin-1; OCLN, occludin; TJP1, tight junction protein 1; JAK, Janus kinase; STAT1, signal transducer and activator of transcription 1; ALT, alanine aminotransferase; AST, aspartate aminotransferase; MDR, multidrug resistance; GI, gastrointestinal; LGG, *Lactobacillus rhamnosus* GG.Bold text highlights the principal mechanistic findings and key concepts for each mycotoxin, facilitating comparison across toxins.

The intestinal epithelium regulates luminal–systemic exchange through tight junction (TJ) complexes. Mycotoxins compromise this barrier by altering TJ protein expression and increasing paracellular permeability, for example, in epithelial cell models exposed to patulin and other mycotoxins (Kolodziej et al., 2011).

Barrier disruption permits microbial products, such as lipopolysaccharide (LPS) to enter underlying tissues, activating toll-like receptor 4 (TLR4) signalling through focal adhesion kinase, interleukin-1 receptor (IL-1R) associated kinases and myeloid differentiation primary response 88 (MyD88), and promoting persistent low-grade inflammation (Zeisel et al., 2005; Guo et al., 2015; Gao et al., 2020). Mechanistically, these changes could interact with microbial detoxification pathways forming feedback loops between epithelial damage and toxin transformation by microbes (Mafe and Büsselberg, 2026).

### 2.1 Aflatoxin B1

Aflatoxin B1 (AFB1) illustrates how epithelial injury intersects with microbiome-dependent immune dysfunction. AFB1 is classified as a Group 1 human carcinogen by the International Agency for Research on Cancer (Iarc, 2002) and contaminates maize, peanuts, tree nuts and animal feeds in warm climates, entering dairy products as aflatoxin M1 (AFM1) after livestock metabolism. Beyond its established carcinogenicity, AFB1 also exerts systemic immunotoxic effects (Gong et al., 2016; Benkerroum, 2020) that depend partly on gut microbial composition and metabolism (Chałaśkiewicz et al., 2025).

Multiple microbial taxa degrade AFB1 through reductive and oxidative ring-modifying pathways distinct from hepatic Cytochrome P450 (CYP450) bioactivation. Key enzymatic activities include laccase-mediated oxidation (e.g., from *Bacillus licheniformis* and *Streptomyces* spp.), F420H₂-dependent reductases from mycobacteria, peroxidase-mediated degradation (e.g., from *Pleurotus ostreatus* and *Phanerochaete sordida*), and glutathione S-transferase-mediated conjugation (Eshelli et al., 2015; Yao et al., 2021; Chen et al., 2022; Rahman et al., 2025; Söylemez et al., 2025; Zhang and Yang, 2025). The genes encoding these activities have been identified in metagenomic and functional screens, but their expression and activity in the gut environment remain poorly characterized.

In mice, antibiotic-mediated microbiota depletion (ABX; antibiotic-induced depletion of gut microbial communities) eliminated AFB1-induced splenic pyroptosis, demonstrating that intact microbiota are required for this immunopathological response (Chen et al., 2024a). Conversely, faecal microbiota transplantation (FMT; transfer of donor microbial communities) and sterile faecal filtrate transfer recapitulated hepatic pyroptosis, implicating gut-derived pipecolic acid and norepinephrine as causal microbial mediators (Ye et al., 2024a). Consistent with these findings, earlier studies reported microbiome-dependent inflammatory liver injury in AFB1-exposed rodents (Liu et al., 2023; Ye et al., 2024a, Ye et al., 2024b), highlighting the gut microbiome as a determinant of systemic mycotoxin toxicity.

In mice, AFB1 reduces faecal short-chain fatty acids (SCFAs) while increasing cholic acid, linoleic acid and pyruvate, indicating alterations in gut microbial metabolism (Zhou et al., 2018). Depletion of the SFCAs butyrate and propionate impairs regulation of inflammatory T-cell responses and enhances nuclear factor kappa-light-chain-enhancer of activated B cells (NF-κB) signalling (Zhang et al., 2021; Akhtar et al., 2022), thus, promoting systemic inflammation. Reduced butyrate availability also compromises mitochondrial function. Conversely, butyrate supplementation restores Parkin (PRKN)-dependent mitophagy, decreases hepatic TLR4 expression, and suppress macrophage activation, ultimately attenuating liver inflammation (Pant et al., 2023).

Recent studies further implicate inflammasome pathways in AFB1-associated toxicity. Increased intestinal permeability facilitates the translocation of LPS, which activates TLR4 → MyD88 → NF-κB signalling cascade, providing the priming signal for NOD-like receptor family pyrin domain-containing 3 (NLRP3) inflammasome (Zhang and Yang, 2023; Yang et al., 2025). In addition, dysbiosis-associated metabolites, including pipecolic acid and norepinephrine, can further stimulate TLR4 and NLRP3 signalling, resulting in caspase-1 activation, cleavage of gasdermin D (GSDMD), and the subsequent maturation and release of the pro-inflammatory cytokines interleukin-1β (IL-1β) and interleukin-18 (IL-18) (Wu et al., 2019; Zhang and Yang, 2023).

*In vitro* digestion models indicate that AFB1 undergoes partial microbial transformation during gastric and duodenal phases, particularly by SCFA–producing lactic acid bacteria, whereas dysbiosis reduces this capacity and increases absorption of residual toxin in the small intestine (Min et al., 2021; Sobral et al., 2022; Rafai et al., 2025). Several microbial taxa, including probiotic strains, metabolize AFB1 via reductive and ring-modifying pathways that diverge fundamentally from hepatic CP450-dependent biotransformation (Eshelli et al., 2015; Yao et al., 2021; Chen et al., 2022; Rahman et al., 2025; Söylemez et al., 2025; Zhang and Yang, 2025)., thereby avoiding epoxidation into the DNA-reactive aflatoxin B1−8,9−epoxide (AFBO) and hydroxylation into AFM1 (Wang et al., 2022). The distinction between microbial and hepatic biotransformation is crucial to understanding local intestinal versus systemic toxicity. However, direct evidence for specific gut microbes mediating AFB1 detoxification *in vivo* remains limited. Microbiota from black soldier fly larvae degrade AFB1 (Yuan et al., 2025), and studies in *Caenorhabditis elegans* indicate that bacterial pyruvate metabolism influences AFB1 toxicity more strongly than host metabolism (Tang et al., 2023). In rodents, *Lactobacillus casei* Shirota partially mitigates AFB1-induced microbiota shifts (Liew et al., 2019), and in ruminants, ruminal microbes convert a fraction of AFB1 to aflatoxicol (Upadhaya et al., 2010). Together, these findings indicate that microbial activity modifies host responses to AFB1, although mechanistic evidence in mammals remains limited. Furthermore, it is important to note that high *in vitro* degradation or binding rates by probiotics do not necessarily persist *in vivo* because of gastric pH, transit time, competing food matrices, host metabolism, and enterohepatic recirculation (Guerre, 2020).

### 2.2 Ochratoxin A

Ochratoxin A (OTA) shows a distinct exposure pattern but similar effects on intestinal barrier function. OTA, classified as a Group 2B possible human carcinogen, occurs widely in cereals, dried fruits, coffee, wine, cocoa and spices in both temperate and tropical regions, particularly during post-harvest storage and processing. OTA therefore poses sustained dietary risk owing to its chemical stability, long biological half-life, renal accumulation and persistence at low exposure levels (Bennett and Klich, 2003; Mally et al., 2005; EFSA Panel on Contaminants in the Food Chain (CONTAM) et al., 2020; Li et al., 2021a; Gemede, 2025; Mubarik et al., 2025).

Microbial detoxification of OTA, primarily via carboxypeptidase− and peptidase−mediated hydrolysis of OTA to OTα together with cell−wall−mediated binding, substantially reduces OTA intestinal bioavailability and attenuates gut−derived liver injury *in vivo* and during food fermentation. Fermented dairy and cereal consortia, particularly Tibetan kefir communities enriched in *Lactobacillus kefiranofaciens* and *Kazachstania* species alongside diverse lactic acid bacteria and yeasts, bind and degrade OTA through multivalent interactions involving cell wall β-glucans, polysaccharides and peptidoglycans (Taheur et al., 2017; Du et al., 2021; Du et al., 2022; Owolabi et al., 2022). However, the extent to which these processes occur *in vivo*, particularly under conditions of microbiota disruption, remains incompletely characterised.

In the intestinal barrier, OTA disrupts epithelial integrity by altering the expression and cellular localisation of TJ proteins, reducing transepithelial electrical resistance and increasing paracellular permeability. Mechanistically, OTA downregulates claudin-1 (CLDN1), occludin (OCLN) and zonula occludens-1 (ZO-1) through reactive oxygen species (ROS), calcium and myosin light chain kinase (MLCK)-dependent signalling. OTA also induces mitochondrial ROS production, leading to caspase-dependent apoptosis and further compromising epithelial barrier function (Wang et al., 2018; Moonwiriyakit et al., 2023). The resulting increase in intestinal permeability promotes mucosal inflammation, characterised by elevated production of interleukin-6 (IL-6), interleukin-1β (IL-1β), tumour necrosis factor-α (TNF-α) and interferon-γ (IFN-γ), accompanied by increased macrophage infiltration and enhanced T helper 17 (Th17) cell responses (Grebenciucova and VanHaerents, 2023; Kappari et al., 2024; Mubarik et al., 2025).

OTA also alters microbial metabolism by reducing tryptophan-metabolising taxa and decreasing the concentrations of microbial metabolites such as kynurenic acid and indole-3-acetic acid (Ma et al., 2023). This results in weaker aryl hydrocarbon receptor (AhR) signalling and regulatory T cell function compromising intestinal immune homeostasis. In parallel, OTA suppresses mitochondrial adenosine triphosphate (ATP) and nicotinamide adenine dinucleotide (NAD^+^) production, activating AMP-activated protein kinase (AMPK) and autophagy, further contributing to epithelial dysfunction. Loss of barrier disruption facilitates dysbiosis-derived LPS into the portal circulation to the liver, where it activates TLR4 signalling and exacerbates hepatic inflammation and permeability (Bennett and Klich, 2003; Turner, 2009).

### 2.3 Fumonisin B1

Fumonisin B1 (FB1), the most toxicologically relevant fumonisin, is prevalent in maize and maize-derived products due to contamination by *Fusarium* species. Exposure causes equine leukoencephalomalacia (LEM) and is associated with increased oesophageal cancer incidence in populations with high maize consumption (Early, 2009; EFSA Panel on Contaminants in the Food Chain (CONTAM) et al., 2018; Mukherjee et al., 2021). FB1 competitively inhibits ceramide synthase (CerS), disrupting *de novo* sphingolipid biosynthesis and causing accumulation of free sphingoid bases, which drive downstream metabolic dysfunction and cytotoxicity (Merrill et al., 1993; Riley et al., 1996; Tsunoda et al., 1998). This mechanism underlies the characteristic increase in sphinganine-to-sphingosine ratios used as a biomarker of exposure (Merrill et al., 1993; Riley et al., 1993; Tran et al., 2006; Voss and Riley, 2013; Zhang et al., 2024b).

Beyond sphingolipid disruption, FB1 also impairs intestinal barrier integrity, induces oxidative stress, activates inflammatory signalling and alters hepatic metabolism (Riley and Merrill, 2019; Cao et al., 2022, Cao et al., 2025; Li et al., 2022; Zhang et al., 2024b). During alkaline processing or microbial metabolism, FB1 is hydrolysed fumonisin B1 (HFB1) by removal of tricarballylic acid side chains. This biotransformation reduces CerS inhibition and sphingolipid disruption, thereby attenuating intestinal and hepatic toxicity in animal models (Maragos et al., 1996; Voss et al., 2009; Grenier et al., 2012). However, HFB1 retains biological activity and can sustain intestinal and hepatic injury through MAPK and NF-κB dependent signalling pathways (He et al., 2006; Masching et al., 2016; Schertz et al., 2018; Chlebicz and Śliżewska, 2020).

Gut-associated lactobacilli contribute to FB1 detoxification through esterase- and amidase-mediated hydrolysis. Among the best-characterised bacteria, *Lactobacillus pontis*, *L. amylovorus* and *L. ultunensis*, isolated from porcine caecum, converted FB1 to HFB1 via surface adsorption followed by enzymatic hydrolysis, achieving up to ~50% conversion within 48 h *in vitro* (Dang et al., 2025). Earlier studies also demonstrated detoxification in broilers using *Lactobacillus plantarum* MYS6 (Deepthi et al., 2017). More broadly, the efficiency of microbial fumonisin detoxification varies substantially among bacterial species and strains, reflecting differences in esterase and amidase activities and limiting the predictability and translational application of probiotic interventions (Voss et al., 2009; Wang et al., 2023).

Although some acylated HFB1 derivatives retain *in vitro* activity, HFB1 generally shows lower *in vivo* toxicity than FB1 (Skácel et al., 2021). This discrepancy reflects the distinction between direct target engagement in cell-based systems and whole-organism determinants of toxicity, including pharmacokinetics, tissue distribution and metabolic clearance (Seiferlein et al., 2007; Sutherland et al., 2012). Removal of the tricarballylic acid chain increases intestinal bioavailability relative to FB1 (Caloni et al., 2002). However, toxicokinetic studies in poultry indicate faster systemic clearance, contributing to reduced overall toxic burden despite its greater absorption (Masching et al., 2016; Gu et al., 2019; Antonissen et al., 2020; Ye et al., 2025). Similarly, studies in rats have shown distinct elimination kinetics for FB1, HFB1 and FB1–fructose conjugates, highlighting compound-specific metabolic handling (Hopmans et al., 1997; Dantzer et al., 1999). In pigs, despite the relatively low oral bioavailability of FB1, systemic absorption leads to accumulation in the liver and kidneys, while sub-chronic exposure disrupts intestinal barrier integrity, immune function and microbial succession without overt enterotoxicity, indicating substantial perturbation of gut ecosystem homeostasis at environmentally relevant doses (He et al., 2006; Hahn et al., 2015; Mateos et al., 2018).

Comparative animal studies consistently demonstrate that both FB1 and HFB1 induce intestinal and hepatic injury, including epithelial damage, villus atrophy, inflammatory cell infiltration and reduced SCFA production, although these effects are generally less severe following HFB1 exposure (Kappari et al., 2024; Ye et al., 2025). Overall, although HFB1 no longer strongly inhibits ceramide synthase and does not elevate sphinganine-to-sphingosine ratios, it retains measurable intestinal and hepatic toxicity, HFB1 still induces milder inflammatory responses. Mechanistically, both compounds compromise epithelial barrier integrity, alter gut microbial composition, reduce SCFA production and disrupt hepatic metabolism primarily through MAPK- and NF-κB-dependent pathways, largely independent of sphingolipid disruption (He et al., 2006; Kim et al., 2015; Gu et al., 2019; Li et al., 2021a, Li et al., 2021b; Qu et al., 2022; Yu et al., 2022b). Thus, current evidence supports a model in which fumonisin toxicity reflects coordinated disruption of epithelial barrier function, gut microbial ecology, immune signalling and hepatic metabolism rather than solely defective sphingolipid biosynthesis (Lázaro et al., 2024; Ye et al., 2025).

### 2.4 Trichothecenes

Another major class of *Fusarium*-derived mycotoxins comprises the trichothecenes deoxynivalenol (DON) and T-2 toxin (T-2). DON is the most prevalent globally, contaminating wheat, maize, barley and oats, whereas T-2, although less frequent, poses heightened risk during outbreaks and under climate-driven shifts in *Fusarium* ecology (Munkvold et al., 2019; Ekwomadu et al., 2021; Janik et al., 2021; Okorski et al., 2022; Kos et al., 2023).

DON exerts toxicity primarily through inhibition of translation, disruption of epithelial barrier integrity and induction of inflammatory signalling. It activates ribotoxic stress pathways that phosphorylate eukaryotic translation initiation factor 2 alpha 9 (eIF2α) and engage MAPK and NF-κB cascades, leading to cytokine induction and intestinal dysfunction, including altered TJ proteins such as ZO-1 and claudins (Pinton et al., 2009; Zhai et al., 2022; Chen et al., 2024b). *In vivo*, DON reshapes intestinal microbial communities and compromises barrier function, linking dysbiosis to impaired growth performance and host resilience in poultry (Lucke et al., 2018; Chen et al., 2024b). These effects were partially mitigated by probiotic interventions, including selected strains of *Bacillus velezensis*, which restored tight junction protein expression, suppressed TLR4/NF-κB signalling and normalised microbial composition and SCFA profiles (Bai et al., 2023; Liang et al., 2024; Wang et al., 2024; Huang et al., 2026). Consistent with these *in vivo* findings, epithelial monolayer studies showed that DON reduces transepithelial electrical resistance and increases paracellular flux, confirming direct disruption of tight junction integrity (Pinton et al., 2009, Pinton et al., 2010; De Walle et al., 2010; Zhang et al., 2022). Although *Bacillus subtilis* strains can enhance epithelial integrity and dampen inflammatory responses, these effects were strain-dependent and not specific to DON exposure. Interestingly, *in vitro* studies also demonstrated that DON and ZEN are effectively deconjugated by the human colonic microbiota, releasing their toxic aglycones and generating yet unidentified catabolites (Dall’Erta et al., 2013).

T-2 similarly induces systemic immunotoxicity through inhibition of protein synthesis and epithelial barrier disruption. T−2 has been shown to disrupt the intestinal microbiota, impair normal intestinal metabolic function and damage intestinal epithelial cells through oxidative stress, inflammatory responses and apoptosis (Zhang et al., 2024a). Liu et al. (2025) showed that T-2 toxin-induced splenic injury is mediated by microbiota-dependent activation interleukin-6 (IL-6)/Janus kinase (JAK)/signal transducer and activator of transcription 1 (STAT1) signalling, as the phenotype was abolished by ABX and recapitulated by faecal FMT. This inflammatory response was attenuated by elemental nano-selenium, suggesting that targeting microbiome–immune interactions may represent a strategy to reduce T-2 toxicity (Wu et al., 2020; Liu et al., 2025). These findings extend earlier evidence implicating JAK/STAT-driven apoptosis and pro-inflammatory gene expression as core mechanisms of trichothecene toxicity (Wang et al., 2012). Consistently, T-2 exposure in ducks induces ileal microbiota dysbiosis and barrier dysfunction, promoting LPS translocation, hepatic TLR4 activation and dysregulation of lipid metabolism genes (ANGPTL4, CPT1B, PLIN1) amplifying inflammatory cascades and lipid accumulation (An et al., 2025). In contrast to other mycotoxins, evidence for binding-mediated sequestration of trichothecenes is limited, suggesting that biotransformation rather than adsorption is the dominant microbial mechanism influencing their toxicity.

## 3 Mycotoxin-driven modulation of gut microbial dynamics and antimicrobial resistance

The human gut microbiota comprises ~10^13^–10^14^ bacterial cells, dominated by Firmicutes (L*actobacillus, Clostridium, Faecalibacterium*) and Bacteroidetes (*Bacteroides, Prevotella*), with contributions from Actinobacteria, Proteobacteria, and Verrucomicrobia; low-abundance taxa such as *Bifidobacterium* and *Escherichia* are maintained under healthy conditions (Gilbert et al., 2018; Liew et al., 2019; Guerre, 2020; Hu and Shen, 2024). Mycotoxins function as microbial substrates and ecological stressors, imposing selective pressures that restructure communities, alter metabolic outputs, and modulate host physiology.

**Figure 1:**
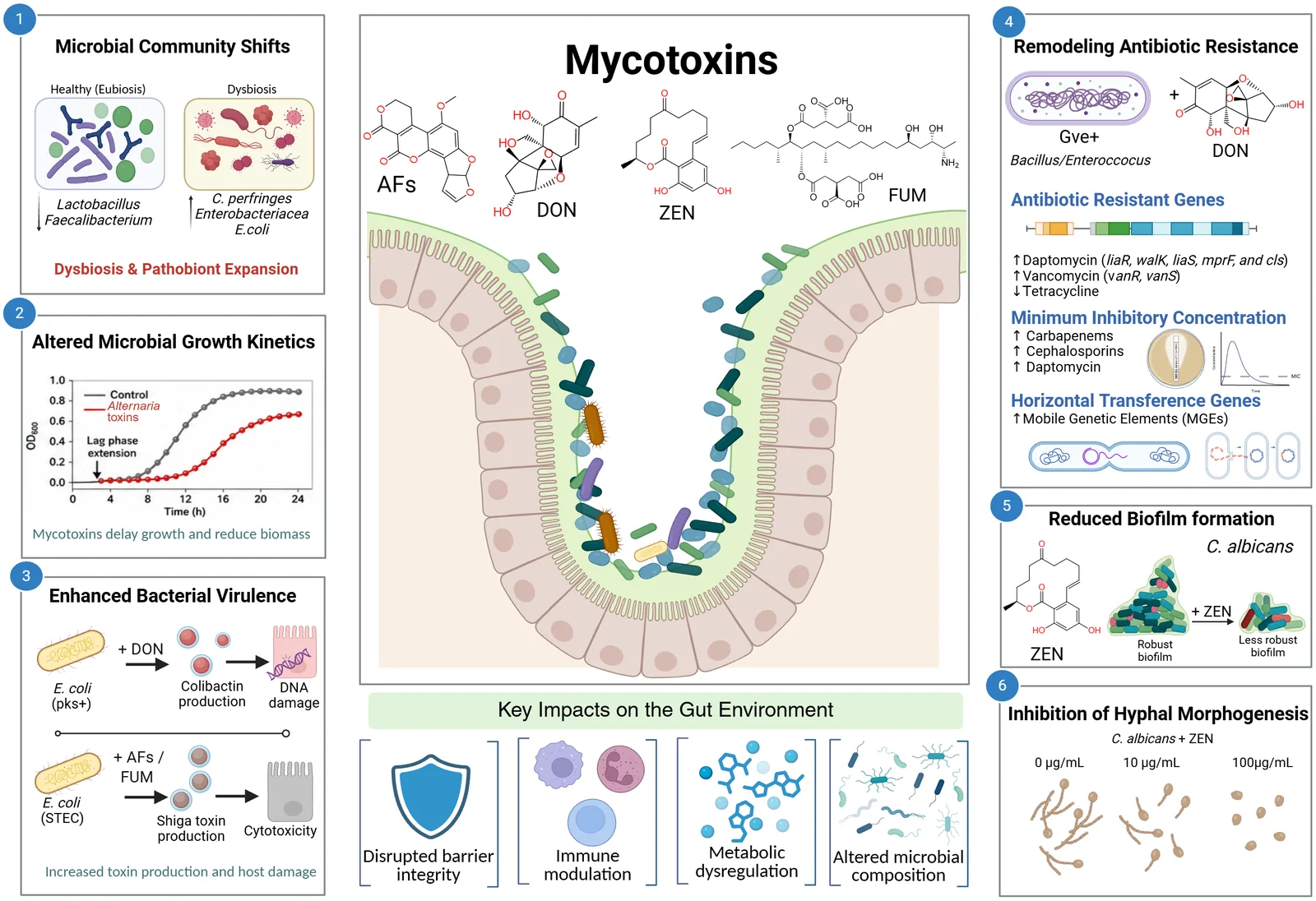
Mechanisms by which mycotoxins influence gut microbiota and host interactions. Schematic representation of the impact of major mycotoxins on gut microbiota composition, function, and host interactions. (1) Mycotoxin exposure induces microbial dysbiosis, characterised by increased abundance of pathobionts (e.g., *Clostridium perfringens* and Enterobacteriaceae, including *Escherichia coli*) and alterations in beneficial taxa. (2) Microbial growth kinetics are affected, including prolonged lag phases (Crudo et al., 2021). (3) Bacterial virulence is enhanced, with increased toxin production and genotoxic effects in pathogenic *E. coli*. (4) Mycotoxins interact with the intestinal epithelium, influencing barrier integrity and host–microbe interactions. (5) Antibiotic resistance profiles are remodelled, including changes in resistance gene expression, increased minimum inhibitory concentrations, and enhanced horizontal gene transfer via mobile genetic elements. (6) Biofilm formation is reduced. (7) Fungal morphogenesis is inhibited, including reduced hyphal formation in *Candida albicans* (Created with BioRender.com). AFs, aflatoxins; DON, deoxynivalenol; ZEN, zearalenone; FUM, fumonisins; MIC, minimum inhibitory concentration; DNA, deoxyribonucleic acid; Gve+, Gram-positive bacteria; Gve−, Gram-negative bacteria; STEC, Shiga toxin-producing *E. coli*; *C. albicans*, *Candida albicans*; *E. coli*, *Escherichia coli*.

Mechanistically, mycotoxins often apply taxon-specific inhibitory effects, such that sensitive populations decline while more tolerant taxa expand. However, reported microbiota responses to mycotoxin exposure remain inconsistent, with DON, ZEN and AFB1 producing opposing shifts in Proteobacteria, Firmicutes and Bacteroidetes across experimental systems. Such variability likely reflects differences in host biology, exposure intensity, exposure duration and matrix composition, underscoring that mycotoxin–microbiome interactions are shaped by ecological context and pre-existing community structure. Results for *Lactobacillus* are particularly inconsistent, despite its relevance in mycotoxin detoxification. Opportunistic pathogens such as *Escherichia coli, Clostridium perfringens*, and *Salmonella* may also increase depending on toxin type and dose in animal models, indicating a shift toward pathobiont dominance. Changes in key taxa, including *Coprococcus, Prevotella*, and *Ruminococcaceae*, further suggest a restructuring of microbial networks (Liew and Mohd-Redzwan, 2018; Jin et al., 2021; Xia et al., 2022a) (**Figure 1**). These compositional shifts translate into functional consequences: mycotoxins can potentiate pathogen virulence and host damage, as exemplified by DON-enhanced genotoxicity of colibactin-producing *E. coli* and increased abundance and cytotoxicity of Shiga toxin-producing strains following AFs and FUM exposure (Baines et al., 2013; Payros et al., 2017). Biofilm formation, central to gut microbial stability and stress tolerance, can also be modulated by mycotoxins. For instance, ZEN reduces *Candida* biofilm formation (Lingueglia, 2018; Rajasekharan et al., 2018).

*Alternaria* mycotoxins can show differential effects on Gram-positive (Gve+) and Gram-negative (Gve-) bacteria *in vitro*, but there is limited experimental evidence: *Clostridium innocuum* (Gve+) and *Bacteroides vulgatus* (Gve-) are inhibited at 50–100 µg/mL, whereas other Gve- taxa such as *Bacteroides thetaiotaomicron* and *Escherichia coli* tolerate higher concentrations (around 200 µg/mL) and accumulate alternariol derivatives likely reflecting differences in membrane composition and lipid partitioning (Crudo et al., 2021). At sub-inhibitory concentrations, mycotoxins such as DON (0.5–50 µg/mL) alter bacterial growth kinetics by prolonging lag phase and reducing growth rates, thereby reshaping competitive dynamics without inducing overt lethality. Notably, these adaptive responses overlap with recognised stress pathways activated by antibiotics, including those associated with fluoroquinolone-induced growth delay (Theophel et al., 2014).

In this context, DON has emerged as a potential ecological driver of antimicrobial resistance. DON exposure selectively enriches Gve+ taxa with antibiotic resistance genes, especially those associated with last-resort antibiotics like daptomycin. It also increases reactive oxygen species production and membrane permeability, amplifying resistance phenotypes (Deng et al., 2025, Deng et al., 2026). *Enterococcus faecalis* isolated from DON-exposed microbiota showed extensive drug resistance, with resistance exceeding 128 µg/mL to daptomycin, vancomycin and linezolid. DON-induced stress promotes genomic plasticity and enhances the co-occurrence and dissemination of antibiotic resistance genes (ARGs) via mobile genetic elements. Bacterial defence responses to DON also converged with classical antibiotic resistance mechanisms, including activation of ATP-binding cassette transporters, toxin-detoxifying enzymes and stress-response proteins like porins, glutathione S-transferases and phosphotransferases (Hassan et al., 2019). These mechanistic parallels should, however, be interpreted cautiously because the evidence base spans different model systems and experimental contexts. Thus, DON is not an antimicrobial in the conventional sense but may act as a selective ecological pressure that favours resistant populations and increases the abundance and mobility of ARGs (Deng et al., 2025). Furthermore, evidence that AFB1, OTA or ZEN directly promote horizontal ARG transfer remains limited.

## 4 Final remarks

Mycotoxins are now recognised as important modulators of gut health, yet the mechanistic basis of their effects on epithelial barrier integrity, immune responses and microbial ecology remains incompletely resolved. A key question is the identity and role of microbial taxa that persist or expand under mycotoxin exposure. For example, aflatoxin exposure can increase *Lactobacillus* populations, are typically considered beneficial and capable of contributing to mycotoxin detoxification. This raises mechanistic questions about whether microbial enrichment reflects adaptive detoxification, ecological resilience to toxin−induced stress, or selection for intrinsically resistant taxa with altered functional potential. Are observed compositional shifts protective or compensatory responses to dysbiosis and barrier disruption?

Mycotoxins may also increase microbial virulence and pathogenicity, causing more damage to the host. This emphasises the importance of considering toxin exposure’s impact on disease-related functional traits. However, many experiments use high doses and non-human models, raising questions about their relevance to real-world conditions, where exposure is usually chronic and low-dose and may allow microbial communities to adapt and develop tolerance or resistance to mycotoxin stress. Addressing these gaps will require a shift beyond descriptive microbiota profiling toward integrative frameworks that link microbial ecology, host physiology and metabolomic outputs. Priority areas include *in vivo* validation of microbial detoxification pathways, systematic comparison of *in vitro* and dietary exposure models, and strain-level functional characterisation to resolve inter- and intra-species variability in detoxification capacity. Multi-strain consortia, including kefir-like communities, are particularly attractive because they can couple adsorption, biodegradation and ecological competition within a single system. Looking ahead, engineered microbial systems could be designed to express defined detoxification enzymes or high-affinity binding modules, but their deployment will require careful evaluation of containment, regulatory approval and ecological impact. Future studies must incorporate co-exposure scenarios, enterohepatic cycling and host-specific variables to enhance translational relevance. Mechanistic resolution of feedback loops between epithelial injury and microbial metabolic reprogramming will be essential to understand whether host–microbiome interactions amplify or constrain toxicity over time. These approaches suggest that the next generation of interventions will likely be modular, mechanism-based and tailored to the toxin, matrix and host context rather than dependent on a single “probiotic” solution.
